# “Moving between living in the shadow of pain and living a life with the pain in the shadows” – women’s experiences of daily life with chronic widespread pain: a qualitative study

**DOI:** 10.1080/17482631.2021.1926057

**Published:** 2021-05-14

**Authors:** Malin Westergården, Katarina Aili, Ingrid Larsson

**Affiliations:** a School of Health and Welfare, Halmstad University, Halmstad, Sweden; b Spenshult Research and Development Centre, Halmstad, Sweden; c Unit of Occupational Medicine, Institute of Environmental Medicine, Karolinska Institutet, Stockholm, Sweden; d Department of Clinical Sciences, Section of Rheumatology, Lund University, Lund, Sweden

**Keywords:** Chronic widespread pain (CWP), daily life, experiences, qualitative content analysis, women

## Abstract

**Purpose**: Long-term pain is a public health problem but few studies have focused on experiences among women with CWP. This study aimed to explore women’s experiences of the impact of CWP on daily life.

**Method**: The participants were 19 women between 45–67 years old, who had developed CWP between 1995 and 2016. Individual interviews were conducted and analysed with qualitative content analysis.

**Results**: Daily life with CWP was expressed in the main theme “Moving between living in the shadow of pain or living a life with the pain in the shadows” including three themes and eight subthemes; 1) living with invisible challenges by feeling neglected as a person and feeling lonely among other people; 2) struggling with limitations by moving between ability and inability, stress and worries, and being dependent on others; and 3) encountering daily life with varying degrees of flexibility by standing still and giving up, moving back and forth by adapting and striving forward with resistance.

**Conclusions**: Women experienced different ways of how CWP influenced their daily life with challenges, limitations, and flexibility. Daily life with CWP entails moving between living in the shadow of pain and living a life with the pain in the shadows.

## Background

About 10% of the general population has chronic widespread pain (CWP) and the condition is more common among women (Mansfield et al., 2016). Some factors that increase the risk of CWP are increasing age, being a woman, living in a socio-economically disadvantaged area, or being born abroad (Bergman et al., 2002).

Pain is defined according to The International Association for the Study of Pain (IASP) as, “*An unpleasant sensory and emotional experience associated with actual or potential tissue damage, or described in terms of such damage”* (Loeser & Treede, 2008, p. 475). Long-term pain, or chronic pain, can be described as when the pain lasts longer than the time the injury is expected to heal within, usually after three months (Treede et al., 2015). Chronic widespread pain (CWP) is commonly defined in epidemiological studies by the American College of Rheumatology (ACR) 1990 criterion for fibromyalgia, where pain should be present in both sides (left and right) of the body, above and below the waist, and in the axial skeleton (Wolfe et al., 1990).

Living with long-term pain influences life in several aspects. As a result of this, people living with long-term pain have a major impact on society including healthcare services costs, thus, generating considerable costs to society. The costs apply to both indirect and direct costs in form of loss of production, unemployment, and increased care-related appointments in healthcare (Henschke et al., 2015). The severity of the pain is seen as a clear factor in relation to which activities can be performed and where help from other family members is seen as crucial to function on a daily basis (Bunzli et al., 2013; Toye et al., 2017). However, a difference is seen when the pain increases, which can contribute to increased isolation and guilt because the ability to perform activities with other family members is reduced (Bunzli et al., 2013; Froud et al., 2014). The experience of living with chronic pain has been described in a number of studies (Froud et al., 2014; Snelgrove & Liossi, 2013; Toye et al., 2017). Although CWP is more common in women than men (Mansfield et al., 2016), there is a lack of studies focusing solely on women with CWP. However, women with long-term pain state that they feel responsible for meeting external demands and expectations from society, regarding work duties, family, and household chores, despite experiencing simultaneous internal pain (Samulowitz et al., 2018). It also appears that both healthcare professionals and the public show distrust and negative attitudes towards women with pain conditions. For example, women living with long-term pain often experience their credibility being questioned through a nonchalant attitude from healthcare professionals in terms of them being perceived as outwardly healthy (Samulowitz et al., 2018).

Pain is perceived and affects the daily life of women with CWP in different ways. Different models have tried to explain the life transition to chronic disease. Gullacksen and Lidbeck (2004) propose the model for life adjustment describing the complex process of change in life. In the model, turning points and passages are described in three stages concerning the past, present, and future. In the initial process, people struggle to restore daily life as it was prior to the development of chronic pain. This is followed by feelings of sorrow and loss, but new understandings and coping skills gradually develop. The process of adjustment ultimately progresses into the establishment of a new course of life. (Gullacksen & Lidbeck, 2004). Another model that explains the life transition is the fear-avoidance model describing the risk of a downward spiral when an injury has occurred with consequences such as pain-related fear, avoidance, and disability. The person experiences an increased disability due to the injury, where negative perceptions can lead to avoidance and abstaining from activities that are believed to increase the pain. Avoiding activities in daily life can give rise to negative thoughts and emotions such as sadness and frustration (Vlaeyen & Linton, 2000). The need for adaptation to the pain continues during life, and to face the pain, as opposed to avoiding the pain, is a core feature of rehabilitation (Gullacksen & Lidbeck, 2004; Vlaeyen & Linton, 2000).

Long-term studies have shown that CWP can only be regarded as persistent for approximately 50% of the population (Landmark et al., 2019), which suggests that half of the individuals reporting CWP at a specific point in time will fluctuate between CWP, and chronic pain in several pain sites but not fulfil the criteria for CWP, or no chronic pain over time. Female gender is one of the factors that predict the persistence of CWP (Mogard et al., 2019; Mundal et al., 2014). Among women, the prevalence of chronic pain seems to increase the most in the age group between 35 and 50 years old (Bergman et al., 2001), suggesting a potentially increased vulnerability for chronic pain and CWP during middle age in women. There is a lack of studies that focus on experiences in daily life in women with CWP, where daily life reflects their current state and reality.

A “chronic pain condition” is a very broad description of a condition that represents a heterogeneous group of individuals suffering from rather different symptoms. Although CWP has a clearer definition, as argued above there are reasons to believe that the individuals who fulfil the criterion for CWP at a specific point in time are also rather heterogeneous in the presentation of their condition. A conscious selection of study participants for studies aiming to explore a specific pain condition may thus be beneficial.

Different models of how chronic pain influences life, and how life influences chronic pain have been described e.g., by the fear-avoidance model and the life adjustment process. However, few studies have described the variations in how middle-aged women with CWP experience this phenomenon from a daily life perspective. The aim of this study was thus to explore different experiences of how daily life is influenced by CWP among women.

## Methods

### Design

The study had an explorative design, based on a latent qualitative content analysis with an inductive approach. The method aims to identify variations in the participant’s subjective experiences in the text from transcribed interviews and then process these by condensation, coding, and categorization (Graneheim & Lundman, 2004). The four aspects of trustworthiness in qualitative studies, credibility, dependability, confirmability, and transferability are taken into account (Graneheim et al., 2017; Graneheim & Lundman, 2004) and the study is reported in accordance with the Consolidated Criteria for Reporting Qualitative Research (COREQ) 32-item checklist (Tong et al., 2007).

### Participants and setting

The participants have been recruited from the EPIPAIN cohort (n = 3928) with men and women aged 20–74 years from the general populations from two municipalities in southwest Sweden. The EPIPAIN study was conducted in 1995 to investigate the risk factors and prevalence of musculoskeletal long-term pain and data were collected with follow-ups in 1998, 2003, 2007, and 2016 (Bergman et al., 2002).

A purposive sample of participants from the EPIPAIN cohort was conducted to achieve a variety of possible outcomes, based on age, education, pain sites, civil status, and employment (Table I). For this qualitative study, women aged 45–67 who had not reported CWP in the questionnaire in 1995 but who had reported CWP according to the ACR 1990 criterion for fibromyalgia (Wolfe et al., 1990) in the follow-up questionnaires in 2016 were eligible. A total of 92 women were eligible according to the criteria. Information on the purpose of the study and the offer for participation was thereafter sent to 43 women, of whom 19 aged 45–67 (median 57 years) accepted to participate in the study, The remaining 15 women chose not to respond and nine women declined to participate in the study. Those who declined did not give further explanations.

**Table I.** Details of the 19 participating women

		Median (range)	N(%)
**Age**, Median (range)		57(45–67)	
**Education**			
	Primary school		2(11)
	Secondary school		12(63)
	College/university		5(26)
**Pain sites**		8(4–16)	
**Civil status**			
	Married/cohabitant		14(74)
	Single		5(26)
**Employment**			
	Working full or part-time		13(68)
	On sick leave		3(16)
	Retired		3(16)

### Data collection

Data collection took place from May to November 2017. The interviews with the participants were conducted in Swedish individually by the authors KA or IL, who previously did not know the participants. The interviews began with open questions so that all participants would have equal opportunities to answer the same questions (Kvale & Brinkmann, 2014). The focus of the interviews was on women’s experience of living with CWP. The questions were; What does pain mean for you? How do you experience your pain? Can you describe your experiences of living with pain? How does pain influence your life today? To encourage participants to develop the answers, follow-up questions were used, such as “how do you mean?” or “what do you have in mind when you say … ?” Two pilot interviews were conducted to check the questions, and no revision of the questions was deemed to be needed. All the interviews, including the two pilot interviews, are included in the analysis. The interviews were digitally recorded and lasted between 25–96 minutes, with a median of 59 minutes. The total interview time was 18 hours and 35 minutes. The interviews were transcribed verbatim.

### Data analysis

A qualitative content analysis according to Graneheim et al. (Graneheim et al., 2017) was used for the data analysis. The purpose of applying a qualitative content analysis was to describe an interpretive process focusing on the subject and the context, as well as dealing with variations in differences and similarities within or between parts of the text (Krippendorff, 2013). The text was read through repeatedly to gain an overall view of the content. The text intended for the aim of the study was subsequently extracted, which constituted the *unit of analysis*. The text was then condensed, resulting in 218 *meaning units*, which were abstracted and then coded. The *codes* were interpreted according to differences and similarities and categorized into *subthemes* and then *themes*. An example based on the analysis schedule is described below, where a code becomes a subtheme and then a theme. The code was *People don´t trust me because the pain is not visible*. The subtheme was then, *feeling neglected as a person* and the theme became, *living with invisible challenges*. A total of eight subthemes and three themes corresponded to the purpose and meaning of the interviews. The underlying meaning of the context was formulated and described as the *main theme* in the study (Graneheim & Lundman, 2004). To increase trustworthiness, the authors, who all had experiences of working in healthcare, continuously discussed all parts of the material during the process until consensus was reached.

### Ethical considerations

Ethical principles underlying the study are based on the Declaration of Helsinki (WMA, 2013) concerning medical research on people and national guidelines on ethical principles. The study was approved by the Ethical Review Board in Lund, Sweden (Reg. No. LU 389–94, 2016/132, 2016/786). The study follows requirements concerning research, consent, information, safety, and confidentiality for participants, based on ethical principles such as non-maleficence, justice, and autonomy (Swedish Research Council, 2017). Participants received oral and written information about the study, that participation was voluntary and that they could decline to continue their participation without giving a reason. Data collection was obtained confidentially and in decoded form and keys, where information about the participants was handled separately, and which only the researchers in this project had access to.

## Results

The participants experienced that daily life was influenced by CWP in terms of moving between living in the shadow of pain and living a life with the pain in the shadows. This meant that the attitude towards the pain varied between allowing the pain to overshadow the daily life and adapting life to the pain, or resisting the pain to set the terms to be able to live a satisfying life. There was a variation to which extent the pain overshadowed daily life, where the participants with CWP were living with invisible challenges, struggling with limitations, and encountering daily life with varying degrees of flexibility (Table II).

**Table II.** Overview of the main theme, themes, and subthemes constructed from the qualitative content analysis of women’s experiences of daily life with CWP (*n*= 19)

The main theme	*Moving between living in the shadow of pain and living a life with the pain in the shadow*		
Themes	**Living with invisible challenges**	**Struggling with limitations**	**Encountering daily life with varying degrees of flexibility**
Subthemes	Feeling neglected as a person	Moving between ability and inability	Standing still and giving up
	Feeling lonely among other people	Struggling with stress and worry	Moving back and forth by adapting
		Being dependent on others	Striving forward with resistance

### Living with invisible challenges

The theme, *living with invisible challenges* included the two subthemes: feeling neglected as a person and feeling lonely among other people.

#### Feeling neglected as a person

The participants with CWP felt that they were being neglected by healthcare professionals as well as the authorities. Their condition was often not taken seriously because the pain was not visible. Not being taken seriously entailed that the pain increased for some participants, where the feeling of a lack of understanding and nonchalance towards their situation led to a stronger perception of alienation in their daily life. The participants felt mistrusted when contacting healthcare professionals or the authorities. One participant stated,

*“You get sad when you don’t feel you’re trusted by the healthcare services and I sometimes think that it’s my fault … //Pain is not visible”*. [P 17]

#### Feeling lonely among other people

Feeling of loneliness among other people meant that they lacked being part of the social context of daily life. Some participants still felt loneliness due to the pain, despite having people around them in social gatherings. Emotions such as anger, sadness, and bitterness as a result of their situation meant that they would rather avoid social contexts and be cut off from reality. The ignorance of the public regarding the pain-affected daily life to such an extent that most participants described that they no longer managed to meet with their friends when the pain was at its worst. Some participants stated that they no longer participated in social activities due to fear of being negatively judged by other people, despite knowing how pleasurable they had previously experienced being in a social context. One participant expressed loneliness,

*“Previously, when people invited me to parties or to meet people, I have abstained due to the pain … //So now people are tired of this, so I’m not now invited anywhere, except by the family. I’ve lost a lot of friends and social … //I didn’t think about it before when the pain was at its worst. I don’t know if I’ve done something wrong in these situations”*. [P 18]

### Struggling with limitations

This theme, *struggling with limitations* contains the three subthemes, moving between ability and inability, struggling with stress and worry, and being dependent on others.

#### Moving between ability and inability

Participants with CWP felt that the pain affected their possibilities of carrying out daily activities, such as household chores, gardening, or duties related to work. The ability to act differed between participants, where the movement between ability and inability varied. Performing daily activities with the family or travelling were also seen as difficult as the ability or inability to move unhindered was affected by the pain. Some of the participants chose not to tell people around them about the pain because they did not want to be judged about their ability or inability due to the pain, even though their pain increased when performing the activity. The participants also felt that their ability to sleep and recover was suffering due to the pain. Not being able to perform daily activities because of not getting sufficient sleep as a result of the pain caused frustration, where the participants’ lives with their relatives were also adversely affected. One participant explained that living with pain included limitations such as being able to be close and intimate with her husband, which had a negative effect on their life together,

*“Not being able to be intimate with my husband has affected our relationship negatively, having physical contact increases the pain and it’s something that’s not spoken about … //It’s been very difficult because there’s no opportunity to be there for him when I have such pain”*. [P 11]

#### Struggling with stress and worry

Living with CWP was double-edged, the pain caused increased stress, but the stress also caused more pain for the participants. The pain had an impact on the body, mind, and soul, where they expressed emotions such as sadness, bitterness, and hopelessness about their situation but also worry for the present and the future. The stress also caused increased tension in the body, which resulted in increased pain and fatigue afterwards. Planning was required in order to avoid stressful situations in daily life. Activities that involved spontaneity required more energy and instead caused increased worry and nervousness that allowed for more pain, where the CWP affected their whole situation. One participant stated how the pain affected her,

*“Pain is a discomforting condition that affects more than just the location of the pain, it affects one’s whole situation, concentration, and general disposition”*. [P 8]

#### Being dependent on others

Participants with CWP felt dependent on other people’s support for carrying out activities, including an understanding of their situation from family and friends. This was the most important thing for having a well-functioning daily life without limitations. They described that the pain led to a feeling of increased empathy, humility, and understanding for people who live with chronic conditions, where one helps the other. Dependence on other people means that activities that can be performed unhindered on certain days, can be impossible to do due to the pain on other days. Participants with CWP felt that friends and family had an increased understanding of the consequences that the condition entailed. Having to depend on others was perceived differently by participants with CWP, where one participant stated,

*“The pain comes stealthily and affects everyday things that you can no longer do when you have to wash or go to the toilet … //It comes gradually, so I’ve got used to it. I’ve become much better at asking for help, not having to be able to do it by myself all the time”*. [P 10]

### Encountering daily life with varying degrees of flexibility

The theme, *encountering daily life with varying degrees of flexibility* includes the three subthemes, standing still and giving up, moving back and forth by adapting, and striving forwards with resistance.

#### Standing still and giving up

Living with CWP meant that some participants abstained from activities that increased the pain, including physical activities such as swimming, gardening, or walking but also social activities such as dancing or taking care of grandchildren. The participants stated that fatigue and fear increased the pain and were their reasons for abstaining from these activities. The pain was described as an unwelcome companion, where it felt as though the body was protesting and limiting the mind and causing them to give up due to the activity. The reason for giving up was that the fear of getting worse was greater than the joy that the activity brought to them. One participant stated,

*“The pain affects my social life, I’ve become restricted. I’d like to do more things, dance, walk more often, or swim. I daren’t do it because I’m afraid it’ll get worse”*. [P 16]

#### Moving back and forth by adapting

Other participants with CWP stated that encountering daily life with flexibility was based on the possibility of adapting. The participants found other ways to carry out the activity, where the awareness of being able to take a step back was just as important as moving forward. In order to cope with an activity, the participants chose to plan and structure the task, by finding new ways and solutions, which led to joy when the task was completed. Sharing household chores or gardening for shorter periods to avoid increased pain or finding other positions to work in making it easier for them in their daily lives. Participants living with CWP described that many adaptations in daily life were carried out automatically because the pain was such a large part of their lives. One participant described it as,

*“I probably live as I’ve always done, I don’t live so differently because of the pain … //I make practical adjustments in everyday life, different work activities”*. [P 5]

#### Striving forward with resistance

Striving forward with resistance as a form of flexibility meant resisting the pain that determined the conditions for daily life. This included the participants with CWP challenging themselves to carry out activities that could lead to increased pain as a result of the action. Being mentally strong, i.e., by showing a fighting spirit, and not letting the fear of the pain affect daily life, but instead embracing positive thoughts and looking ahead was described as important because it prevented the pain from prevailing. Refraining from carrying out social activities or physical activities was not described as an alternative, but instead, there was a strategy to perform the activity and not be affected by the pain. One participant expressed,

*“I don’t get less pain just because I think about it but I think based on my experience … //I can’t worry about tomorrow, but instead, I take advantage of the opportunity that comes and make the best of it, that’s my way and I think that I can cope that way”* [P 6]

## Discussion

Moving between living in the shadow of the pain and living a life with the pain in the shadows leads to the impact of CWP differing from one day to the other. The women either allow the pain to overshadow their daily life, adapting their lives to the pain, or resisting the pain to determine the terms of daily life. The result shows that how women experience the impact of CWP on their daily life differs between individuals. Also, their approach to a life in pain, and the need to adjust to the pain differs among the women.

The result shows that middle-aged women with CWP face daily life challenges and limitations with different inherent and external expectations and express different needs for adjustments, or flexibility, in daily life. These different approaches have similarities to the models of fear avoidance and life adjustment (Gullacksen & Lidbeck, 2004; Vlaeyen & Linton, 2000). Avoiding actions in daily life can cause a downward spiral and generate negative thoughts and emotions such as sadness and frustration (Vlaeyen & Linton, 2000). Another approach is the life adjustment process, where the maintenance of living with pain involves adjustments for the future when the pain brings about new demands in daily life (Gullacksen & Lidbeck, 2004). Although the fear-avoidance model and the life-adjustment process can be seen as contrasts, there are elements of the models that overlap, where they include differences in managing to live with a long-term pain condition (Gullacksen & Lidbeck, 2004; Vlaeyen & Linton, 2000). These differences are in line with what was found in this study, where it is described as an ongoing struggle between living in the shadow of pain and living a life with the pain in the shadows. Based on these approaches, daily life with CWP can be described as women’s processes for life adjustment and what approach they have to be flexible, standing still and giving up, moving back and forth by adapting and striving forward with resistance. The results from this study imply that the approach towards life in pain can fluctuate daily, depending on inherent and external expectations, pain status, and motivation.

Standing still and giving up means refraining from activities with the fear that the pain is exacerbated, where negative thoughts and fear for the pain can lead to avoidance behaviour, which generates an increased risk of disability (Vlaeyen & Linton, 2000), social withdrawal (Froud et al., 2014), and depressive symptoms (Snelgrove & Liossi, 2013). Moving back and forth by adapting involves consciously carrying out activities that are in balance with the pain and finding new ways to cope, where an acceptance of the pain can help the individual to attain the ability to move forward (Biguet et al., 2016). Managing life with pain is also described as adjusting to the uncertainty the pain causes (Snelgrove & Liossi, 2013). The pain can change from one day to another, which some of the women in this study automatically adapted to due to the pain being such a central part of their life. The result revealed another form of flexibility when the women respond to the pain with resistance where the pain-inducing activity is performed regardless of the consequences. To be able to challenge oneself before carrying out an activity requires a balance between what the mind wants and what the body is capable of (Gullacksen & Lidbeck, 2004). The result describes some of the women with CWP having an awareness of how the pain may be experienced before the action is carried out, but that the desire to participate in the activity is stronger than the desire to abstain, although the consequence can be increased pain. This study shows that middle-aged women living with CWP should be seen as a heterogeneous group, where they face daily life in different ways. The need for flexibility varied based on a number of different factors, where the uncertainty about pain reflects the complexity of the variation of invisible challenges and limitations in life.

Some of the women in this study spoke of feeling neglected by healthcare professionals, authorities, and by society in general. They expressed that their pain experience was questioned and that they were treated with nonchalance in their quest to get a correct diagnosis. This is in line with the findings of previous research, where it has been reported that people with long-term pain perceive a reduced understanding of their condition from healthcare professionals and society and that their ability to influence their care is limited (Froud et al., 2014; Toye et al., 2013). This is consistent with what was expressed by the women in this study—that one must be strong to be sick.

The women in this study also talked about a feeling of loneliness, although they were together with other people. This feeling of loneliness among other people can reflect stigmatization (Froud et al., 2014). Some of the women in the study kept the extent of their pain to themselves with fear of being judged negatively by people around them. It has previously been described how people with long-term pain experience an increased risk of stigmatization due to the variability of their pain severity. They are able to carry out certain activities one day but have to refrain from them the next, contributing to experiences of people questioning their credibility (Bunzli et al., 2013; Froud et al., 2014). The need for more knowledge on the underlying mechanisms, the sources of the stigmatizing behaviour, and the impact of stigmatization on the individuals has been emphasized and argued for in previous studies (De Ruddere & Craig, 2016). Although the problem is well recognized, the knowledge is too scarce to provide effective interventions (De Ruddere & Craig, 2016).

The women in this study spoke of a feeling of limitations in their daily lives, where their ability to perform physical activity, work duties, household chores, or activities with the family were negatively affected. Activities that are performed easily one day can be seen as impossible the next due to the severity of pain, which limits their possibilities of maintaining an independent lifestyle (Toye et al., 2017). The result highlights the women’s experiences of avoiding stressful situations, and that performing an activity to a greater extent involves planning the activity carefully in advance. An inability to perform activities often includes blaming themselves and reduced self-esteem (Samulowitz et al., 2018). A clear aspect that emerges in the result is the complex link between stress and pain, where increased stress leads to more pain, and pain generates more stress. Stress involves stronger emotions such as sadness, bitterness, and greater anxiety over the present and future, and even depressive symptoms (Henschke et al., 2015; Snelgrove & Liossi, 2013). The ability to work is described as an issue of concern in this study, where misunderstanding of the condition contributes to the uncertainty for the women. The invisibility of the pain causes an increased risk of stigma in the workplace because the diagnosis is not considered as being credible (Bunzli et al., 2013; Froud et al., 2014). The result reveals that being dependent on other people is seen as a common factor in women with CWP, where the possibility of performing activities varies from day to day based on the severity of the pain. Paradoxically, the need for support can also mean that people with long-term pain withdraw from activities with the intention of not burdening their family members (Froud et al., 2014). People with chronic pain often feel that they are inadequate in their relationships with their spouse (Bunzli et al., 2013; Toye et al., 2017), which emerges in this study as a limitation in the relationship when the possibility of closeness is impeded due to pain.

## Strengths and limitations

A qualitative content analysis was applied in this study to explore variations of different experiences among the participants (Graneheim & Lundman, 2004). The study was based on a latent content, where the interpretation was grounded on the experience of living with CWP in daily life among women. The *trustworthiness* of the study was based on four aspects, *credibility, dependability, confirmability, and transferability* (Graneheim et al., 2017; Graneheim & Lundman, 2004).

*Credibility* increased since a purposive selection was made prior to the study to obtain as rich a variation as possible, in which variables such as age, education, civil status, and employment were taken into account. The number of informants, 19, and a total of 218 meaning units was extracted and covered the data (Graneheim & Lundman, 2004). The author (MW) coded and analysed the data while the other authors (KA, IL) acted as co-assessors. The authors continuously discussed all steps throughout the process until consensus was reached. The credibility was also based on the different themes constructed during the analysis process, where the goal was to show differences and similarities without one of the themes being perceived as similar to one of the others, which was discussed between the authors. Examples from the analysis schedule from codes, subthemes, and themes were presented to increase credibility (Graneheim et al., 2017). *Dependability* was strengthened by the fact that the same questions were asked to all participants and that they were encouraged to describe their experiences of living with CWP. All the authors had a background in the health and medical fields, which provided an increased opportunity for understanding the participants’ experiences. The authors’ pre-understanding of the subject can be seen as a potential limitation in the study, in that there is a risk that the authors’ pre-understanding unconsciously affects the direction of the analysis process (Graneheim et al., 2017). However, one strength is that the material has been discussed by all the authors and there is an awareness of the risk of the effect of pre-understanding. Another strength is that the data material has been collected by authors (KA, IL) who had extensive methodological knowledge (Graneheim et al., 2017; Graneheim & Lundman, 2004). Subthemes and themes were compared and reviewed until the final ordering was constructed by all authors.

The *confirmability* was strengthened by the systematic process that was carried out during the analysis procedure (Graneheim & Lundman, 2004), where all the steps in the analysis have been reported and described in the study. The participants’ experiences have, as far as possible, been reported in the result, where quotes have strengthened the perceived variation of living with CWP in daily life. The interview texts were deemed rich and contain a great variety of content.

*Transferability* aims to identify and investigate whether the findings are applicable in another setting (Graneheim et al., 2017; Graneheim & Lundman, 2004). By describing the approach, the procedures, and the analysis transparently and clearly, the result can be perceived to apply to a wider population who live with other long-term pain or chronic conditions that are invisible but affect daily life. This study intentionally only included middle-aged women. This may, on the one hand, be seen as a strength since the intention was to describe variations of experiences of living with a similar condition. On the other hand, it is a limitation since it only allows for a specific population to be heard. Another limitation might be that all data were collected from the same regional area. Providing that the purposive selection in the present study represents the variation within the group of women with CWP, the results could be transferable to a wider population. In order to gain a broader picture, future studies should investigate whether other parts of the population experience a daily life with CWP in similar ways.

## Conclusion

This study shows that there is a variation in the extent to which women with CWP experienced invisible challenges and how they struggled with limitations in daily life. Also, the way to respond to their limitations or invisible challenges differed between women, where their flexibility had an impact on how they handled their CWP. This entailed that their attitude towards the pain varied between allowing the pain to overshadow their daily lives, adapting their lives to the pain, or resisting the pain to set the terms (i.e., moving between living in the shadow of the pain and living a life with the pain in the shadows). More qualitative research on women with long-term pain is needed to support women with CWP to find their counterweight to cope with daily life and at the same time allow the women to tell their story.
